# Quiescent keratocytes fail to repair MMC induced DNA damage leading to the long-term inhibition of myofibroblast differentiation and wound healing

**Published:** 2012-07-04

**Authors:** James V. Jester, Chyong Jy Nien, Vasilis Vasiliou, Donald J. Brown

**Affiliations:** 1 The Gavin Herbert Eye Institute, University of California, Irvine, Irvine, CA; 2 Department of Pharmaceutical Sciences, Skaggs School of Pharmacy and Pharmaceutical Sciences, University of Colorado Denver, Aurora, CO

## Abstract

**Purpose:**

The purpose of this study was to determine the acute and long-term effects of mitomycin C (MMC) on quiescent rabbit corneal keratocytes regarding cell proliferation, myofibroblast differentiation and DNA repair.

**Methods:**

Quiescent keratocytes cultured in serum-free media were exposed to various concentrations of MMC and then treated with transforming growth factor-β (TGFβ). DNA damage was evaluated in both cultured keratocytes and live rabbit eyes following treatment with MMC. The long-term ability of quiescent keratocytes to repair MMC induced damage in vivo was evaluated in rabbits treated with MMC 2 months before 100 μm deep lamellar keratectomy (LK) injury.

**Results:**

MMC significantly blocked TGFβ-induced cell proliferation and myofibroblast differentiation in cultured quiescent keratocytes and altered the transcriptional regulation of macrophage chemotactic protein-1 (MCP-1) and alpha smooth muscle actin (αSMA). MMC also induced phosphorylation of the nuclear histone marker of DNA damage, γH2AX (a member of the H2A histone family), without induction of cell cycle entry or immediate DNA repair measured by Comet assay. In live rabbits, 0.2 mg/ml MMC significantly induced γH2AX nuclear immunostaining (p<0.05) throughout the cornea and corneas receiving 0.2 mg/ml MMC treatment 2 months before LK injury showed complete absence of any corneal scarring.

**Conclusions:**

MMC induces DNA damage to quiescent corneal keratocytes, which remains unrepaired, resulting in abnormal cell replication and gene transcription that leads to long-term effects on corneal repair. Overall these findings suggest that there may be long-term and perhaps permanent consequences to the application of MMC as an anti-fibrotic therapy.

## Introduction

Mitomycin C (MMC) belongs to a family of anti-tumor quinolone antibiotics derived from *Streptomyces caespitosus*, and functions as a powerful bifunctional alkylating agent that induces DNA interstrand crosslinks (ICL). These changes lead to the inhibition of cell replication, altered gene transcription and cell death if not repaired [1,2]. While approved as an anti-cancer chemotherpeutic agent, MMC has recently gained widespread clinical acceptance in the ophthalmic community as an off-label prophylactic anti-fibrotic therapy to prevent corneal scarring and haze following corneal refractive surgery, including photorefractive keratectomy (PRK) and laser assisted in situ keratomileusis (LASIK) [3-5]. Treatment of patients immediately following PRK with 0.2 mg/ml (0.02%) MMC for 12 s to 2 min significantly decreases haze and results in better refractive predictability and improved efficacy for over a year and half after surgery [6-9].

Initial laboratory studies evaluating the effects of MMC in rabbits have shown that inhibition of corneal haze following PRK is associated with a significant reduction of keratocyte cell density [10,11]. Later studies have shown that loss of keratocyte density after MMC treatment is associated with increased keratocyte apoptosis and the absence of myofibroblasts within the wound [12-14]. More recent studies have also shown that adjacent keratocytes fail to enter the cell cycle following PRK with MMC treatment suggesting that MMC blocks cell replication [15]. Furthermore, DNA damage as detected by phosphorylation of H2AX, a member of the H2A nuclear histone family that initiates recruitment of nuclear excision repair endonucleases [16-18], has been identified in the nuclei of corneal endothelial cells in eyes treated with MMC under ex vivo conditions [19]. These data indicate that the effect of MMC on corneal scarring and haze is related to the generation of DNA ICLs leading to defective cell replication and gene expression.

While the molecular mechanism underlying MMC’s effect on corneal scarring and haze remains unknown, previous investigators have proposed that increased MMC-induced keratocyte apoptosis and the profound inhibition of keratocyte and myofibroblast progenitor cell proliferation may play a central role [15]. To more clearly understand the process by which MMC inhibits corneal scarring and haze after PRK, we have evaluated the effects of MMC on quiescent corneal keratocytes. Our findings indicate that MMC induces DNA damage that leads to abnormal myofibroblast differentiation, proliferation and gene transcription. More disturbing is the finding that DNA damage in MMC treated keratocytes leads to long-term effects on myofibroblast differentiation and corneal repair in culture and animal wound healing models. Overall, the findings suggest that there may be long-term consequences to the use of MMC as an anti-fibrotic that have yet to be clinically manifested.

## Methods

### Cell culture studies

Rabbit corneal keratocytes were isolated from rabbit eyes (Pel Freez, Rogers, AR) and cultured under serum-free conditions as previously described [20,21]. Cells were plated at a density of 1–5×10^4^ cells/cm^2^ in polystyrene coated tissue culture dishes (Falcon; Becton Dickinson, Franklin Lakes, NJ) and allowed to attach for 72 h. Cells were then treated with various concentrations of MMC (Calbiochem, La Jolla, CA) for 5 min and then rinsed in serum-free media. Cells were then cultured with and without transforming growth factor-β (TGFβ, 5 ng/ml; Sigma-Aldrich, St. Louis, MO) for various times and evaluated by immunocytochemistry, western blotting, and RT–PCR.

### Immunohistochemistry

Cells were fixed in 2% paraformaldehyde in PBS (pH=7.4), then treated with acetone (−20 °C) for 3 min, rinsed with PBS, blocked with goat serum (dilution 1:10 in PBS; Cappel; MP Biomedicals, Solon, OH) for 30 min at 37 °C and stained with monoclonal antibodies to α-smooth muscle actin (αSMA; Sigma, St. Louis, MO) to measure myofibroblast differentiation, the nuclear cell cycle protein Ki67 (clone MIB-2; Inmunotech, Westbrook, ME) to measure cell cycle entry and γH2AX (Abcam, Cambridge, MA) to assess DNA damage [16-18]. Sections were then stained with FITC conjugated goat anti-mouse IgG (Southern Bioech, Birmingham, AL) and counterstained with Alexa 543 phalloidin and DAPI. Sections were then evaluated using the Nikon Eclipse E600 epifluorescence microscope (Nikon Instruments Inc., Melville, NY).

### Cell proliferation

To assess the effects of MMC on TGFβ induced cell proliferation, keratocytes were cultured in 6-well plates (Falcon) and treated with various concentrations of MMC for 5 min. Cells were then cultured with and without TGFβ and the change in cell number measured as previously describe using a Leica DMIRB Inverted microscope (Leica Microsystems, Werzlar, Germany) and Optronics CCD camera (Goleta, CA) [22]. At least three wells/treament condition were measured and the experiment repeated 3 times.

### Western blotting

Total proteins were solubilized in buffer containing 25 mM Tris-HCl (pH 7.4), 1 mM EDTA, 1 mM EGTA, 10 mM dithiothreitol, 1% sodium dodecyl sulfate (SDS), 5 μg/ml antipain, 5 μg/ml pepstatin A, and 1 mM phenyl methyl sulfonyl fluoride (PMSF). Proteins were electrophoresed on precast 10% Tris-glycine sodium dodecyl sulfate polyacrylamide (SDS–PAGE) gels (Invitrogen, Carlsbad, CA). Proteins were transferred to a polyvinylidene difluoride membranes (Bio-Rad, Hercules,CA), blocked for 2 h with 5% BSA in Tris saline and probed with antibodies to αSMA (Sigma-Aldrich) at 4 °C overnight. Blots were then washed with Tween-20 and Tris-buffered saline (50mM Tris,150mM NaCl and 0.05% Tween-20) and incubated for 1 h with a Cy3 conjugated goat anti-mouse IgG (1:5000; Southern Biotech). Western blots were then scanned at an excitation of 532 nm and emission of 580 nm using a fluorescent imager (FMBIO III; Hitachi-MiraiBio Inc., Alameda, CA).

### RNA extraction and PCR

Cells were washed 2× in cold PBS then directly lysed in RLT buffer (Qiagen, Valencia, CA) and the supernatant recovered. The samples were then passed over QiaShredder columns and then processed over RNeasy columns (both from Qiagen). The RNA was eluted from the silica column with 50 μl water, and the product was analyzed using a NanoDrop Spectrophotometer (Thermo Scientific, Wilmington, DE) to estimate RNA concentrations. The RNA (500 μg) obtained from cultured cells was converted to cDNA using the QuantiTect Reverse Transcription Kit (Qiagen). Reactions were performed for 30 min at 42 °C, and 5 min at 95 °C, followed by cooling to 4 °C. After synthesis, the cDNA from all sources was diluted to equivalent input RNA levels with TE (10 mM Tris/HCl, 1 mM EDTA [pH 7.5]; 10 ng input RNA/μl) and stored at −20 °C until use. cDNA samples were subjected to PCR using specific primers (Table 1). Primers were designed using Primer 3 Internet software program (The Whitehead Institute, Cambridge, MA), and their specificities were confirmed by a BLAST Internet software-assisted search of the non-redundant nucleotide sequence database (National Library of Medicine, Bethesda, MD). Polymerase chain reactions (PCR) were carried out as previously described with 5–25 ng reverse-transcribed RNA and 200 nM forward and reverse primers, in a total volume of 25 ml [23]. For quantitative real time PCR (qPCR), samples were normalized by b2-MG amplification and were amplified using the QuantiTect Syber Green reagents (Qiagen) in triplicate using an MJ Research Opticon Thermal Cycler (Waltham, MA).  PCR controls without reverse transcriptase (water control) or with normal human genomic DNA as a template were routinely negative. PCR controls without reverse transcriptase (water control) were routinely negative. Product specificities were confirmed by DNA sequencing the products.

**Table 1:** RT–PCR Primers.

**Product**	**Forward**	**Reverse**	**GenBank**
α smooth muscle actin	TGTGCTATGTCGCTCTGGAC	CTTCTGCATACGGTCAGCAA	X60732
GAPDH	GAGCTGAACGGGAAACTCAC	CCCTGTTGCTGTAGCCAAAT	NM_001082253
β actin	ATCGTGATGGACTCCGGCGAC	AGCGCCACGTAGCACAGC	NM_001101683
MCP-1	CACCCGGACACCCTCTACTA	GATCCTTGCAAGAACCCTCA	M57440

### Comet assay

DNA repair by corneal fibroblasts and keratocytes was assessed by Comet assay using the protocol previously described by Cordelli et al. [24]. Briefly, slides were prepared by dipping in 1% agarose/PBS and allowing to air dry to a thin film. Cells were harvested 24 h post MMC treatment by scraping in cold PBS, centrifuged at 500× g and the cell pellet re-suspended in PBS to 10^7^ cells/ ml. The cell suspension (10 μl) was quickly mixed with 65 μl 0.7% Low Melting Point agarose/PBS, coverslipped, and the agarose allowed to gel at 4 °C. After gelling, the coverslip was removed and the gel overlaid with an additional 100 μl of 0.7% LMP agarose/PBS and allowed to gel. Slides were then submerged overnight at 4 °C in lysis buffer containing: 2.5M NaCl, 100 mM EDTA, 10 mM Tris/HCl pH 10, 1% Triton X 100 and 10% DMSO. The following morning, the slides were placed in lysis buffer containing 10 mM DTT and allowed to incubate, for 30 min at 4 °C. The slides were then placed under freshly prepared electrophoresis buffer composed of: 300 mM NaOH, 1 mM EDTA, adjusted to pH 13 with HCl and held at 4 °C for 20 min. The samples were then electrophoresed at 4 °C, for 5 min at 25V. The slides were then immersed in 0.3 M sodium acetate in 70% alcohol for 30 min, then fixed in 100% alcohol for 2 h, followed by 70% alcohol for 5 min and then allowed to air dry. The slides were stained by immersion in 2 μg/ml Ethidium Bromide and photographed under a 20× objective. Slides were scored using CometScore software v1.5 (TriTek Corporation, Summerduck, VA).

### Rabbit studies

New Zealand albino rabbits were used in this study and all procedures were approved by the UCI IACUC and conducted in accordance with ARVO Statement for the Use of Animals in Ophthalmic and Vision Research. Prior to injury, all rabbits were anesthetized with intramuscular injections of xylazine (5 mg/Kg) (Anased, Lloyd Laboratories, Shenandoah, IA) and ketamine (22 mg/Kg) (Bioniche Pharma USA LLC, Lake Forest, IL). Tetracaine hydrochloride 0.5% eye drops (Alcon Laboratories Inc., Forth Worth, TX) were instilled into each right eye, and a speculum was used to open the eyelids. After surgery, eyes received topical gentamicin sulfate (Akorn, Buffalo Grove, IL) three times a day for 3 days and intramuscular buprenorphine (Buprenx; 0.1 mg/kg; Reckitt Benckiser Healthcare, Hull, UK) once and as needed for relief of pain.

### DNA damage study

A total of 6 rabbits were used in this study. An 8-mm trephine (Bausch & Lomb, Rochester, NY) was used to mark the central cornea and the epithelium was then removed using a Bard Parker® #11 surgical blade (Becton Dickinson AcuteCare, Franklin Lakes, NJ). Both eyes of each rabbit were then randomly assigned to four groups of 3 eyes each and were treated with either 0.2 mg/ml or 0.02 mg/ml MMC for 15 s or 60 s. Animals were allowed to recover and then sacrificed 3 h after treatment.

### MMC and delayed injury study

A total of 10 rabbits were used in this study. Initially rabbits received a unilateral epithelial scrape injury and then randomly assigned to two group of 5 rabbits each and treated with 0.2 mg/ml MMC for 60 s or vehicle control applied using a surgical sponge as previous described [25]. Animals were allowed to recover for 8 weeks and then received a lamellar keratectomy (LK) injury in the same treated eye using techniques previously reported [26,27]. Rabbits were then followed by in vivo confocal microscopy and then sacrificed 3 months after LK injury.

### In vivo confocal microscopy (CM)

Under anesthesia, animals received a baseline scan and were then followed at various times after injury by in vivo confocal microscopy (CM) to measure epithelial and stromal thickness and haze as described previously [28,29]. All in vivo CM examinations were performed using a tandem scanning confocal microscope (TSCM; Tandem Scanning Corporation, Reston, VA) with a 24× surface-contact objective (numerical aperture, 0.6; working distance, 1.5 mm). One drop of 2.5% hydromethylcellulose (Gonak, Akorn, Buffalo Grove, IL) was placed on the tip of the objective as a coupling gel. All camera settings were kept constant throughout the experiment. For each examination, repeated through focus data sets were obtained from the central corneal region. A total of three to five through-focus data sets for each eye at each time point were collected in the thinnest region of the cornea.

### Ex vivo microscopy

After animals were sacrificed, corneas were fixed by anterior chamber perfusion for 4 min with 2% paraformaldehyde in phosphate-buffered saline, pH 7.4. After perfusion, corneas were dissected and left in 2% paraformaldehyde overnight. For assessment of DNA damage following MMC treatment, fixed corneal tissue was embedded in O.C.T Compound (Tissue-Teck, Sakura Finetek, Torrance, CA) and snap frozen in liquid nitrogen. Blocks were then cut on a Leica CM 1850 cryotome (Leica, Wetzlar, Germany) and stained with antibodies specific of γH2AX for 1 h followed by staining with goat anti-mouse IgG conjugated to FITC for 1 h. Slides were then counter stained with DAPI (300 nM, Invitrogen, Carlsbad, CA) and imaged using a Nikon Eclipse E600 epifluorescent microscope equipped with a Photometrics CoolSnap FX camera (Roper Scientific, Tucson, AZ) and images digitized using Meta Imaging Series software (Molecular Devices, Downington, PA). All images were taken using the same camera settings and exposure time, and all slides were stained on the same day. To quantify the intensity of γH2AX staining over keratocyte nuclei, the DAPI channel was used to define regions of interest over nuclei using the create region tool in the Meta Imaging software. These regions were then applied to the γH2AX channel, and the average pixel intensity over the nuclei measured and recorded. To adjust for differences in staining, the average background image intensity overlying non-nuclear areas was also measured and subtracted from the measured nuclear intensity for each slide. On average, a total of 20 nuclei from each corneal section from each region were measured.

To assess corneal fibrosis, collagen organization of the corneas was visualized by non linear optical imaging of second harmonic generated signals from collagen (SHG). SHG signals to detect collagen were generated using 800-nm infrared femtosecond laser pulses to obtain maximum SHG emission signal at 400 nm [25]. SHG forward-scattered signals passing through the tissue were collected using an 0.8 NA condenser lens with a narrow band-pass filter (400/50) placed in front of the transmission light detector. Backward-scattered SHG signals were detected using the Meta Detector set to collect 390 to 410 nm band width on the Zeiss 510 Meta Laser Scanning Confocal microscope (Carl Zeiss Microscopy GmbH, Jena, Germany). To assess cell density in the same optical plane, corneal blocks were also stained with Syto 59 (Invitrogen), and imaged using laser scanning confocal microscopy.

### Statistical analysis

All results are reported as mean±standard deviation. Differences between treatment groups were assessed by two-way repeated-measures ANOVA and Bonferroni multiple comparisons (Sigma Stat version 3.11; Systat Software Inc., Point Richmond, CA). For the live rabbit studies we used a sample size of five eyes per group, with a power of 0.95 and α=0.05 to detect a 50% reduction in haze. All tissue culture experiments were performed in triplicate and repeated at least three times.

## Results

### Effects of MMC on TGFβ-induced myofibroblast differentiation

**Figure 1:**
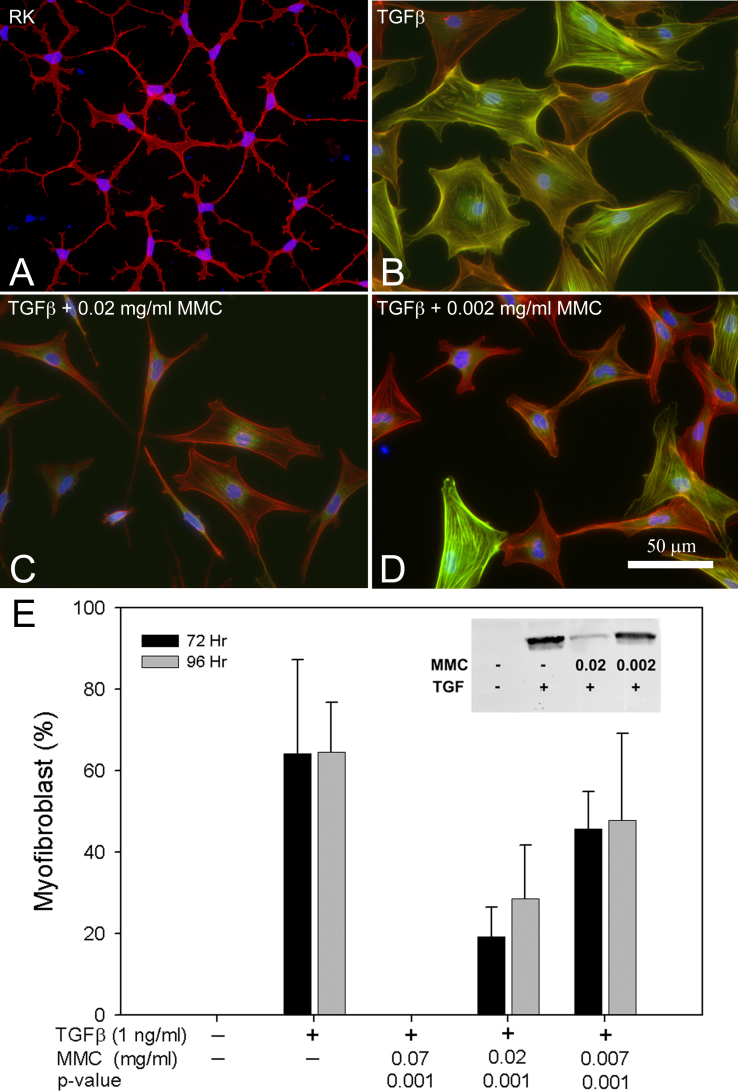
Concentration-dependent effects of MMC on keratocytes treated with TGFβ for 72 h. Cells were untreated (**A**) or treated with TGFβ alone (**B**) or after 0.02 mg/ml MMC (**C**) or 0.002 mg/ml MMC (**D**) treatment and then stained with phalloidin (red), anti-αSMA (green), and DAPI (blue). Cells treated with TGFβ alone showed a spread morphology and αSMA staining typical of myofibroblasts (**B**), while cells treated with MMC before TGFβ showed loss of cell spreading and decreased αSMA staining (**C** and **D**). Quantitation of αSMA staining (**E**) showed significantly reduced numbers of myofibroblasts following pre-treatment with MMC and decreased protein expression for αSMA (insert).

To establish whether MMC blocked myofibroblast differentiation of quiescent keratocytes, cultures were stained with specific antibodies to αSMA, a known biomarker for myofibroblast differentiation [21], and the number of cells expressing αSMA quantified. By comparison, keratocytes cultured under serum-free conditions maintained a dendritic morphology and showed no staining for αSMA (Figure 1A), while cells exposed to TGFβ alone showed cell spreading, increased actin filament assembly and marked expression of αSMA (Figure 1B). When cells were pre-treated with MMC for 5 min and then immediately treated with TGFβ, MMC at concentrations of 0.02 mg/ml and 0.002 mg/ml blocked TGFβ induced cell spreading, actin filament assembly and expression of αSMA at 72 h (Figure 1C,1D). Concentrations above 0.02 mg/ml induced marked keratocyte cell death. Quantifying the number of cells expressing αSMA showed that the pre-treatment of cells with MMC dose dependently and significantly reduced (p<0.001) the number of cells staining for αSMA. Western blots also confirmed the reduction in expression of αSMA after 72 h of TGFβ treatment (Figure 1E, insert). Overall, these data showed that MMC leads to abnormal TGFβ-induced myofibroblast differentiation of rabbit corneal keratocytes.

### Effects of MMC on TGFβ-induced cell replication and gene transcription

**Figure 2:**
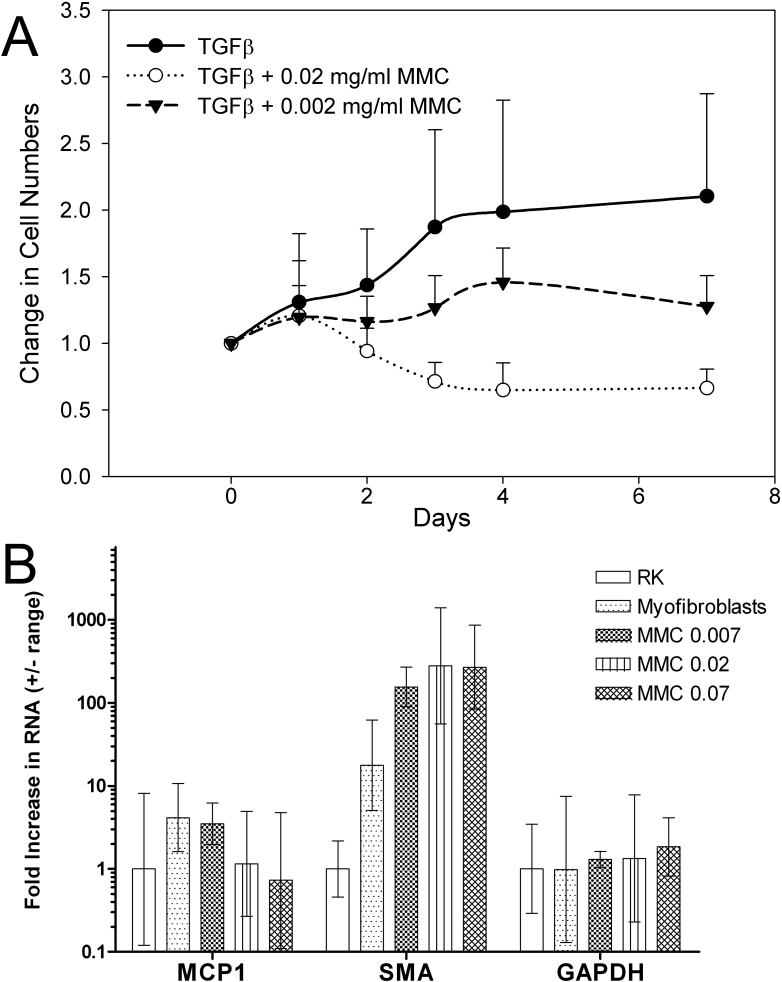
The effects of MMC on TGFβ-induced cell proliferation and gene expression. **A**: The effect of MMC and TGFβ on cell proliferation showed that 0.02 and 0.002 mg/ml significantly reduced (p<0.001) cell growth compared to TGFβ alone. **B**: RT–PCR for *MCP-1* and *αSMA* showed that TGFβ alone induces the expression of both *MCP-1* and *αSMA*, but that MMC dose dependently blocks *MCP-1*, while increasing *αSMA* mRNA when normalized to glyceraldehyde 3-phosphate dehydrogenase (*GAPDH*).

Since DNA damage induced by MMC is known to block cell replication and gene transcription, we next evaluated the effect of MMC on TGFβ-induced cell growth and gene expression of quiescent keratocytes (Figure 2). While keratocytes showed no increase in cell number over 7 days in culture with or without MMC treatment (data not shown), TGFβ treatment alone resulted in a doubling in the cell number (Figure 2A). Pretreatment of cells with 0.02 mg/ml MMC, on the other hand, resulted in a loss of cells averaging only 77%±16% of the original cell numbers 7 days after treatment. Pretreatment with 0.002 mg/ml MMC resulted in only a modest increase in the number of cells over 7 days that remained significantly below TGFβ alone treated cells (p<0.001). When treatment of cells with TGFβ was delayed 7 days to allow for DNA repair, MMC treated keratocytes continued to show a dose dependent decrease in the ability to replicate and express αSMA in response to TGFβ (data not shown) suggesting prolonged effects of MMC that are not repaired in culture.

To assess the effect of MMC on gene expression by quiescent keratocytes, the expression of MCP-1 and αSMA were evaluated by RT–PCR. MCP-1, is a small inducible cytokine belonging to the CC chemokine family that is involved in recruiting macrophages, memory T-cells and dendritic cells to sites of injury and is thought to play an important role in the development of fibrosis [30,31]. As shown in Figure 2B, TGFβ treated quiescent keratocytes showed increased expression of mRNA for *MCP-1* and *αSMA*. Treatment with increasing concentrations of MMC showed a dose dependent loss of TGFβ-induced MCP-1. Interestingly, expression of TGFβ-induced *αSMA* mRNA showed increasing expression with higher concentrations of MMC. Overall, these findings are consistent with MMC induced DNA damage leading to abnormal cell replication and gene expression patterns.

### MMC induced DNA damage and repair in culture

**Figure 3:**
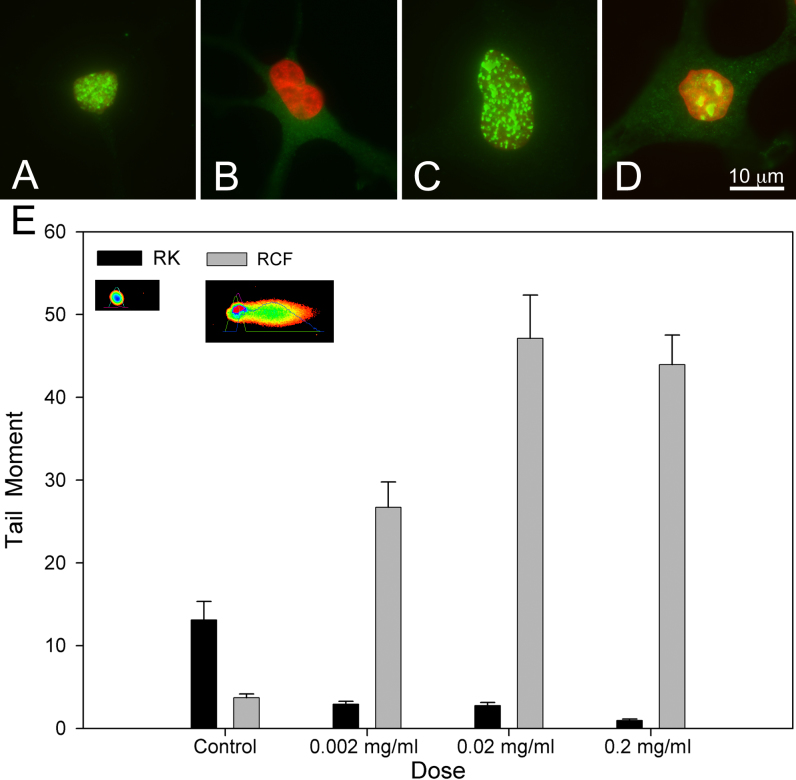
DNA repair in quiescent keratocytes compared to proliferating fibroblasts following MMC exposure. MMC treated Keratocytes (**A** and **B**) and corneal fibroblasts (**C** and **D**) were stained with γH2AX (**A** and **C**, green), Ki67 (**B** and **D**, green) and DAPI (red) 24 h after treatment. Keratocytes showed only γH2AX staining while fibroblasts showed γH2AX and Ki67 staining. Comet assay (**E**) shows that MMC dose dependently increases the Comet tail in fibroblasts 24 h after treatment, while significantly decreasing the Comet tail in quiescent keratocytes.

Since the effects of MMC on quiescent keratocytes were not reversed by extended cell culture, we next evaluated the MMC induced DNA damage and repair. As a bifunctional ankylating agent, MMC induces DNA interstand crosslinks (ICLs) that lead to phosphorylation of histone, H2AX, which recruits nuclear excision repair endonucleases that detach ICLs and repair DNA through a homologous recombination [16-18]. Antibodies specific for γH2AX, the phosphorylated form of H2AX used as a molecular marker for DNA ICLs [16], stained keratocyte nuclei that had been treated with MMC (Figure 3A). Quantification of the number of cells stained by γH2AX following MMC treatment showed a dose dependent increase with concentration of 0.07 mg/ml showing greater than 80% γH2AX staining that peaked 2 days after treatment and decreased to 40% by day 4 (data not shown). When MMC treated keratocytes were stained for Ki67, a marker of cell cycle entry and DNA repair, no Ki67 staining was detected (Figure 3B), suggesting that keratocytes did not go through a normal replication coupled DNA repair pathway. This is different from corneal fibroblasts that showed both γH2AX staining and Ki67 labeling following treatment with MMC (Figure 3C,3D, respectively).

To determine whether MMC treated keratocytes undergo DNA repair, a Comet assay was performed on MMC treated quiescent keratocytes and fibroblast cultures to detect interstrand breaks in nuclear DNA, necessary to uncouple and remove ICLs in preparation for DNA repair. In a Comet assay, DNA migrates from the nuclei, or head of the Comet, and forms a progressively longer tail depending on the number of breaks (Figure 3E, insert). Replicating cells, such as corneal fibroblasts, show a dose dependent increase in the Tail Moment of the Comet when treated with MMC and evaluated 24 h after treatment, indicating uncoupling of ICLs. By comparison, keratocytes showed a marked decrease in Tail moment 24 h after treatment with increasing doses of MMC, suggesting no DNA excision repair following MMC treatment. Overall, these data indicate that MMC dose dependently leads to DNA damage of quiescent corneal keratocytes in culture that does not undergo normal replicative DNA repair.

### MMC induced DNA damage in the cornea

**Figure 4:**
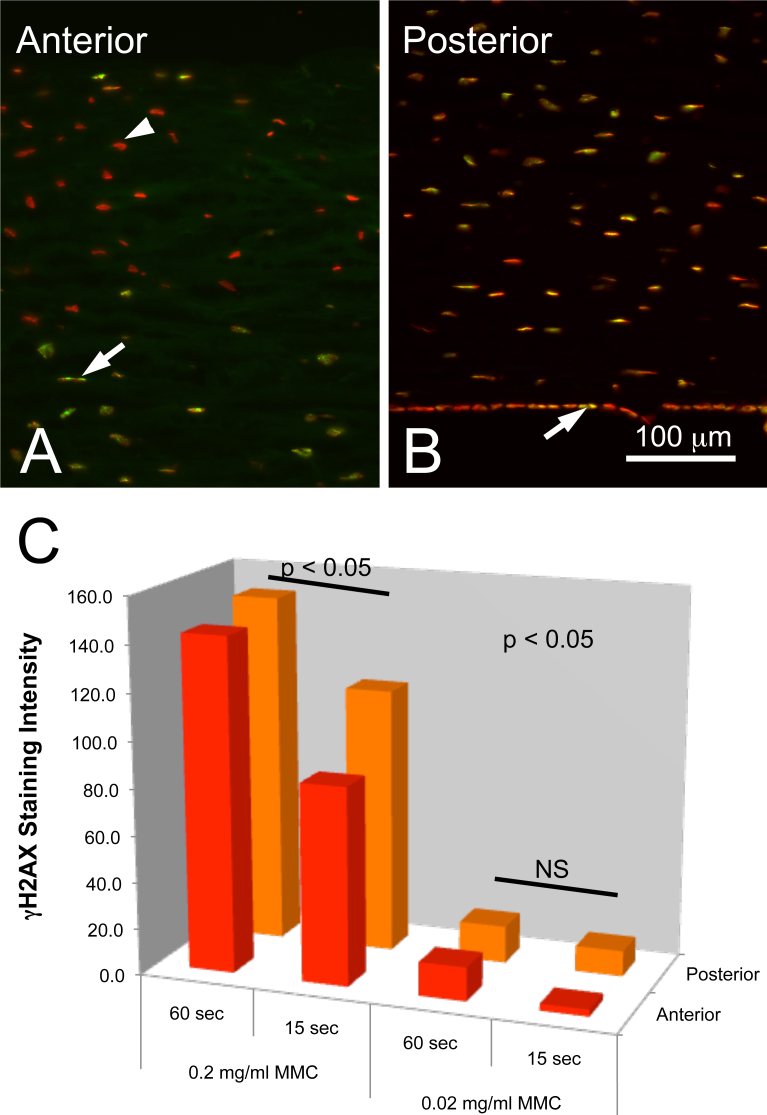
DNA damage in rabbit corneas following treatment with MMC. γH2AX staining (green) and DAPI staining (red) of the anterior (**A**) and posterior (**B**) cornea following MMC treatment. Note that the most anterior keratocytes show only DAPI staining (arrowhead) suggesting apoptosis, while the remaining keratocytes (**A**, arrow) and posterior endothelial cells (**B,** arrow) show γH2AX staining. Quantification of γH2AX staining intensity (**C**) showed that 0.2 mg/ml MMC produced significantly greater staining compared to corneas treated with 0.02 mg/ml, and that 60 s exposure produced significantly greater staining compared to 15 s. It should also be noted that there was no significant difference (NS) in staining between cells in the anterior or posterior cornea.

To determine whether MMC induced DNA damage in the cornea, rabbits were treated with MMC following scrape injury and then evaluated for the expression of γH2AX 3 h after treatment (Figure 4). Sections stained with antibodies to γH2AX (green) and counter stained with nuclear dye (red) showed staining of nuclei both in the anterior stroma (Figure 4A, arrow), posterior stroma (Figure 4B), and corneal endothelium (Figure 4B, arrow). In the very anterior stroma, keratocyte nuclei show little or no staining, most likely related to the known keratocyte apoptosis and cell death following scrape injury (Figure 4A, arrowhead). When the intensity of γH2AX staining over the nuclei was measured, very high intensity was detected over stromal keratocytes when exposed to 0.2 mg/ml MMC for 60 s (Figure 4C). There was a significant decrease in intensity (p<0.05) when the exposure was reduced from 60 s to 15 s, but the intensity of staining remained significantly greater than that detected for corneas treated with 0.02 mg/ml MMC for either 60 s or 15 s, which was not significantly different from background. Regarding differences in staining between anterior and posterior keratocytes, no significant differences were detected for both 60 s and 15 s exposures to 0.2 mg/ml MMC, suggesting all cells in the corneal stroma are equally damaged by MMC treatment in the rabbit at a given dose and exposure. It should also be noted that expression of Ki67 was not detected in corneal sections, consistent with previous studies that have evaluated later time points (data not shown) [15]. Overall, these data indicate that 0.2 mg/ml MMC (either 60 s or 15 s exposure times) is the lowest dose that induces significant DNA damage.

### Effect of delayed injury on MMC treated corneas

**Figure 5:**
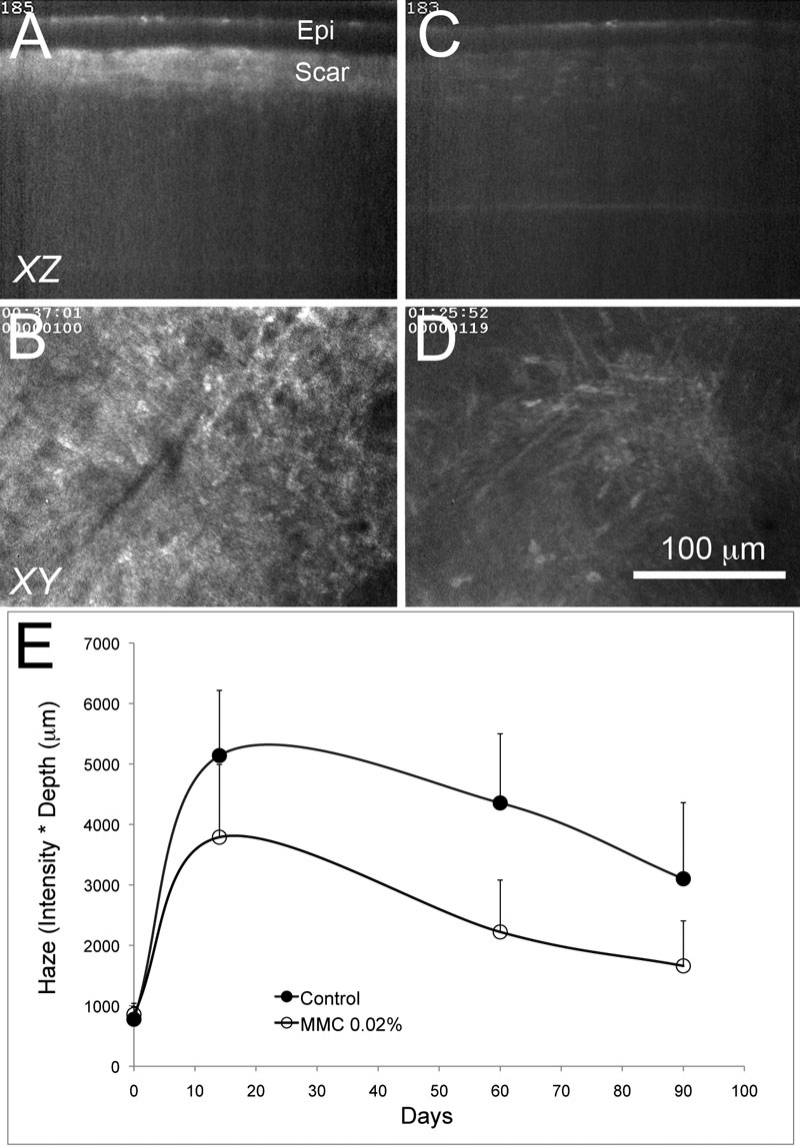
In vivo confocal microscopy of lamellar keratectomy injury in rabbit corneas 2 months after MMC treatment. In vivo confocal microscopy of rabbit corneas receiving vehicle control (**A** and **B**) or 0.2 mg/ml MMC (**C** and **D**) 2 months before lamellar keratectomy (LK). In control eyes, 3 months after LK injury, there is a dense scar underlying the epithelium (Epi) detected in the XZ projection (**A**) that contains densely populated scar fibroblasts seen in the XY plane underlying the epithelium (**B**). Eyes treated 2 months earlier with MMC showed no scar in the XZ projection (**C**) and decreased keratocytes underlying the epithelium in the XY plane (**D**) 3 months after LK injury. Quantification of light scattering showed that eyes treated with MMC 2 months before LK injury had significantly less haze (**E**).

To access repair of MMC DNA damage to quiescent keratocytes in live rabbits, eyes were treated with 0.2 mg/ml MMC for 60 s following epithelial scrape injury. Rabbits were then allowed to recover for 2 months before 100 μm deep LK injury followed by evaluation using in vivo confocal microscopy. As shown in previous studies [26,27], LK injury in control, vehicle treated eyes resulted in significant corneal scarring that was detected by in vivo confocal microscopy as intense light scattering (Scar) underlying the corneal epithelium (Epi) in XZ projections (Figure 5A). In regions of the scar, single planes (XY) showed a high density of scar fibroblasts embedded in highly light scattering extracellular matrix (Figure 5B). By contrast, eyes treated 2 months previously with 0.2 mg/ml MMC showed no corneal scaring in XZ projections (Figure 5C) and in single planes (XY) underlying the corneal epithelium showed a sparse population of keratocytes embedded in low light scattering, transparent extracellular matrix (Figure 5D). Measurement of the light scattering from the corneas also showed significantly reduced (p<0.001) corneal haze in eyes pretreated with 0.2 mg/ml MMC (Figure 5E).

**Figure 6:**
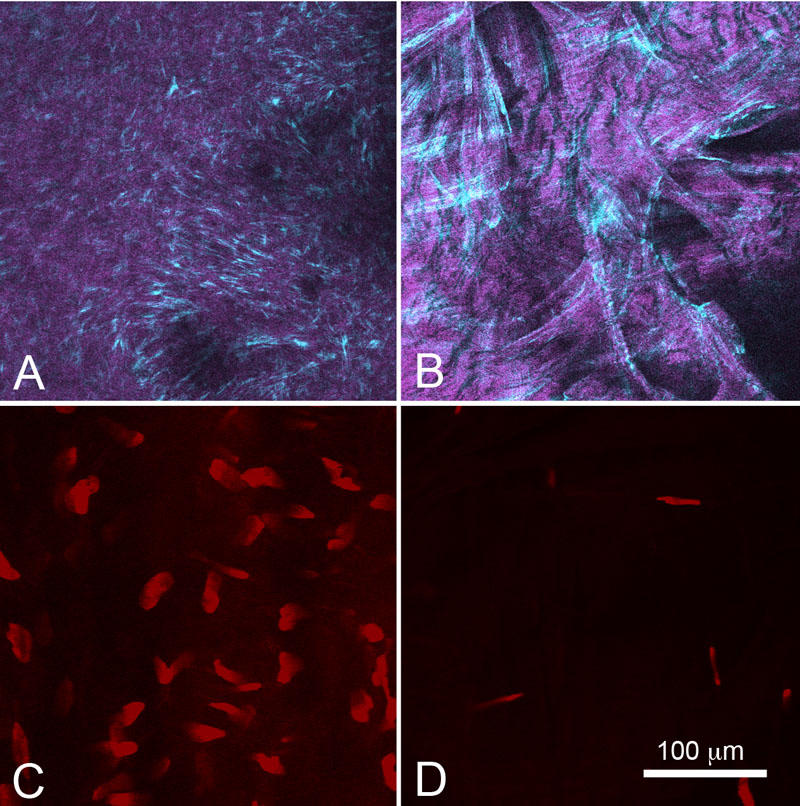
Corneal scarring and keratocyte cell density in rabbit corneas 3 months after lamellar keratectomy in eyes treated with and without MMC. Second harmonic (**A** and **B**) and confocal imaging of the same plane stained with Syto59 (**C** and **D**) of control (**A** and **C**) and 0.2 mg/ml MMC treated corneas (**B** and **D**) 2 months before lamellar keratectomy injury. Control corneas 3 months after LK injury showed the presence of irregularly organized scar collagen (**A**) and a high density of scar fibroblast nuclei (**B**). By comparison, eyes treated with MMC 2 months before LK injury showed normal lamellar stromal collagen at the epithelial-stromal interface (**C**) and markedly decreased corneal keratocytes (**D**) 3 months after LK injury.

The absence of corneal scarring and reduced keratocyte cell numbers was also confirmed by ex vivo confocal and nonlinear optical imaging of collagen using SHG imaging (Figure 6). In vehicle treated eyes, irregular collagen organization was detected by SHG (Figure 6A) that measured on average 84.2±27 μm in thickness. By comparison, eyes previously treated with 0.2 mg/ml MMC showed no irregular collagen deposition and only the presence of normal, broad collagen lamellae at the interface between the corneal epithelium and the stroma (Figure 6B). The nuclear density of the corneal scar in the vehicle treated eyes was also significantly greater (p<0.05) averaging 382±114 cells/mm^2^ immediately underlying the corneal epithelium compared to the MMC treated eyes, which averaged 126±67 cells/mm^2^. Overall, this data suggests that MMC damage to quiescent corneal keratocytes goes unrepaired in rabbits for at least 2 months after treatment.

## Discussion

Previous studies have shown that MMC treatment following PRK results in the induction of keratocyte apoptosis, inhibition of keratocyte proliferation and reduced keratocyte repopulation of the anterior cornea [15,32]. While these findings have lead some investigators to suggest that the apoptotic and anti-proliferative effects of MMC block activation of myofibroblast progenitor cells [33], the mechanism underlying the anti-fibrotic effect of MMC remains unclear. In this report, we show that MMC dose dependently blocks TGFβ-induced proliferation and alters gene transcription of cultured quiescent rabbit keratocytes and that quiescent keratocytes do not recover a normal TGFβ response for at least 7 days in culture. These observations support and extend previous observations in live rabbit eyes and cultured horse corneal fibroblasts and are consistent with the DNA interstrand crosslinking (ICL) effects of MMC [15,34]. More importantly, we show for the first time that the failure of quiescent keratocytes to recover from MMC treatment and respond to TGFβ is associated with failed or greatly delayed DNA repair as evidenced by the absence of cell cycle entry following phosphorylation of H2AX in response to MMC induced ICLs, the prolonged expression of γH2AX indicating the persistence of ICLs, and the lack of formation of DNA interstrand breaks detected by Comet Assay. More troubling was the finding that the effect of MMC persisted for at least 2 months in live rabbits as evidenced by the absence of corneal fibrosis in response to LK injury. Together these findings suggest that the anti-fibrotic effect of MMC is due to persistent DNA damage that blocks the ability of quiescent keratocytes to respond to wound cytokines. While it is not known whether a similar mechanism underlies the anti-fibrotic effect of MMC in patients receiving refractive surgery, these findings suggest that there may be significant long-term consequences for MMC therapy.

Recently, studies evaluating the effects of MMC on the goat cornea have shown that topical treatment with therapeutic doses of MMC induced DNA damage detected by modified Comet assay as increased ‘head’ DNA, consistent with decreased Tail moment measured in this report [19]. Additionally, cultured goat corneal endothelial cells treated with various concentrations of MMC showed persistent γH2AX staining suggesting markedly delayed or defective DNA repair. Importantly, these findings are similar to our findings in the rabbit keratocyte showing both prolonged γH2AX staining and failed TGFβ responsiveness, even after 7 days of recovery in culture. Our in vivo findings that γH2AX uniformly and equally stains anterior and posterior stromal keratocytes as well as corneal endothelial cells, also support the previous observations that the corneal endothelium is a likely target for MMC-induced DNA damage even in thicker human corneas. Furthermore, the suspicion that DNA repair is lacking in the cornea based on prolonged γH2AX staining is confirmed by our finding that 2 months recovery following MMC treatment failed to restore a normal wound healing response in the rabbit.

While DNA repair mechanisms in the cornea are unknown, it is generally thought that cells enter the cell cycle following MMC treatment and that cycle progression at S-phase is blocked, where upon cells undergo DNA repair [35]. In cell cycling competent cells, nuclear excision repair endonucleases (NER) have been shown to ‘unhook’ and remove ICLs, followed by homologous recombination. However, studies of terminally differentiated cells, including muscle and nerve, have shown that this normal repair mechanism is turned off [36]. As an alternative, quiescent, serum-starved cells are thought to have transcription-coupled repair (TCR) mechanisms, acting only on the transcribed genes, and non-transcribed strand repair (NTSR) that act on non-transcribed genes, both through different mechanisms [37]. More recent studies of terminally differentiated muscle cells suggest that these alternative mechanisms are impaired or severely compromised and that accumulation of DNA damage may lead to restricted apoptosis and autophagy [36,38]. Such a fate for MMC treated corneal keratocytes and endothelial cells is suggested by the finding of persistent annexin V staining in goat corneal endothelial cells [19], and the decrease keratocyte density detected in vivo and in culture following TGFβ treatment. Based on this data, additional investigation into the long-term effects of MMC in culture and live animals seems warranted. On a more fundamental level, a better understanding of DNA repair mechanisms of corneal keratocytes and endothelial cells is needed since these cells are exposed to DNA damaging UV and oxidative stress through out life. Importantly, these studies need to evaluate DNA repair mechanisms in quiescent and differentiated cells that critically lack the global genome repair mechanisms that have been extensively studied in serum cultured, cycling cells.

An additional finding in this study was the observation that the only dose of MMC to cause significant γH2AX staining of corneal keratocytes and corneal endothelial cells was the currently accepted therapeutic dose. While there were significant differences in the intensity γH2AX staining comparing 60 s exposure to 15 s exposure, a lower dose of MMC at either 60 s or 15 s exposure failed to show significantly detectible γH2AX staining. Previous studies have extensively evaluated the effect of lower doses of MMC on corneal keratocyte density, cell cycling and haze, and observed some anti-fibrotic effects using a “prophylactic” dose of 0.002% MMC [15]. It should be noted, however, that this study also used a longer exposure interval of 2 min, and that this dose has been shown clinically to produce ‘breakout haze’ [5,15,39]. Studies performed in our laboratory that tested different doses of MMC with exposure times of 15 s found that the only dose that significantly reduced corneal fibrosis as measured by SHG imaging in rabbits was the recognized therapeutic dose, consistent with the γH2AX staining and the clinical observations (data not shown).

In summary, this report provides evidence that DNA damage induced by MMC goes unrepaired in rabbit keratocytes. This finding suggests that DNA repair mechanisms in corneal keratocytes may be limited, and that further studies are needed to evaluate how keratocytes repair DNA damage in the cornea. Since accumulated, unrepaired DNA damage is thought to underlie many degenerative neurologic disorders [40], understanding DNA repair in the cornea may have wider implications than just for MMC-induced corneal injury. While it is widely recognized that the safety of using MMC as an off-label anti-fibrotic has yet to be established, our findings taken together with those of Roh et al. [19] suggest that the lack of DNA repair following MMC treatment could have important long-term consequences in those patients having refractive surgery. Besides the obvious long-term effects on wound healing following accidental injury or additional surgical intervention, the accumulative genomic and mitochondrial DNA damage induced by MMC may increase the susceptibility of patients to other degenerative corneal disorders. Clearly, caution in the use of MMC is warranted until a clearer understanding of the potential long-term side effects have been established.
